# The role of epigenetics in rare diseases

**DOI:** 10.1515/medgen-2024-2014

**Published:** 2024-06-06

**Authors:** Bernhard Horsthemke

**Affiliations:** University Hospital Essen Institute for Human Genetics Hufelandstr. 55 45147 Essen Germany

**Keywords:** Epigenetics, Chromatin, X inactivation, Imprinting, Epimutations

## Abstract

Epigenetic control systems are based on chromatin modifications (DNA methylation, histone modifications and nucleosome positioning), which affect the local kinetics of gene expression. They play an important role in maintaining cell fate decisions, X inactivation and genomic imprinting. Aberrant chromatin states that are associated with a deleterious change in gene expression are called epimutations. An epimutation can be a primary epimutation that has occurred in the absence of any genetic change or a secondary epimutation that results from a mutation of a *cis*-acting regulatory element or *trans*-acting factor. Epimutations may play a causative role in disease, for example in imprinting disorders, or may be part of the pathogenetic mechanism as in the fragile X syndrome and in syndromes caused by a mutation affecting a chromatin modifier. For several diseases, DNA methylation testing is an important tool in the diagnostic work-up of patients.

## Introduction

Many genetic syndromes arise from errors in cell differentiation and embryonic development. Cell fate decisions and cell lineage identity are determined by transcription factors, which activate or repress specific genes, and are stabilized by enzymes that modify chromatin and the local kinetics of gene expression. The major chromatin modifications are the methylation of cytosine residues within CpG dinucleotides of the DNA, histone modifications (mainly acetylation, methylation, phosphorylation and ubiquitination of specific amino acid side chains) and nucleosome positioning. These modifications and the proteins that make, read and erase them are known as “epigenetic control system”. In several cases such as X-inactivation, long non-coding RNAs are also involved. While the term “epigenetics” was coined by Conrad Hal Waddington in 1942 to describe the role of genes in development [1], the term “epigenetic control system” was introduced in 1958 by David Nanney [2], although at that time the molecular mechanisms were unknown. Nanney chose the term “epigenetic” to “emphasize the reliance of these systems on the genetic systems and to underscore their significance in developmental processes”. Most importantly, he pointed out that “certain patterns of expression, although specifically induced, may be perpetuated in the absence of the inducing conditions”. According to Nanney, such a “cellular memory” ensures that differentiated cells maintain their phenotype even through multiple rounds of cell divisions.

**Figure 1:**
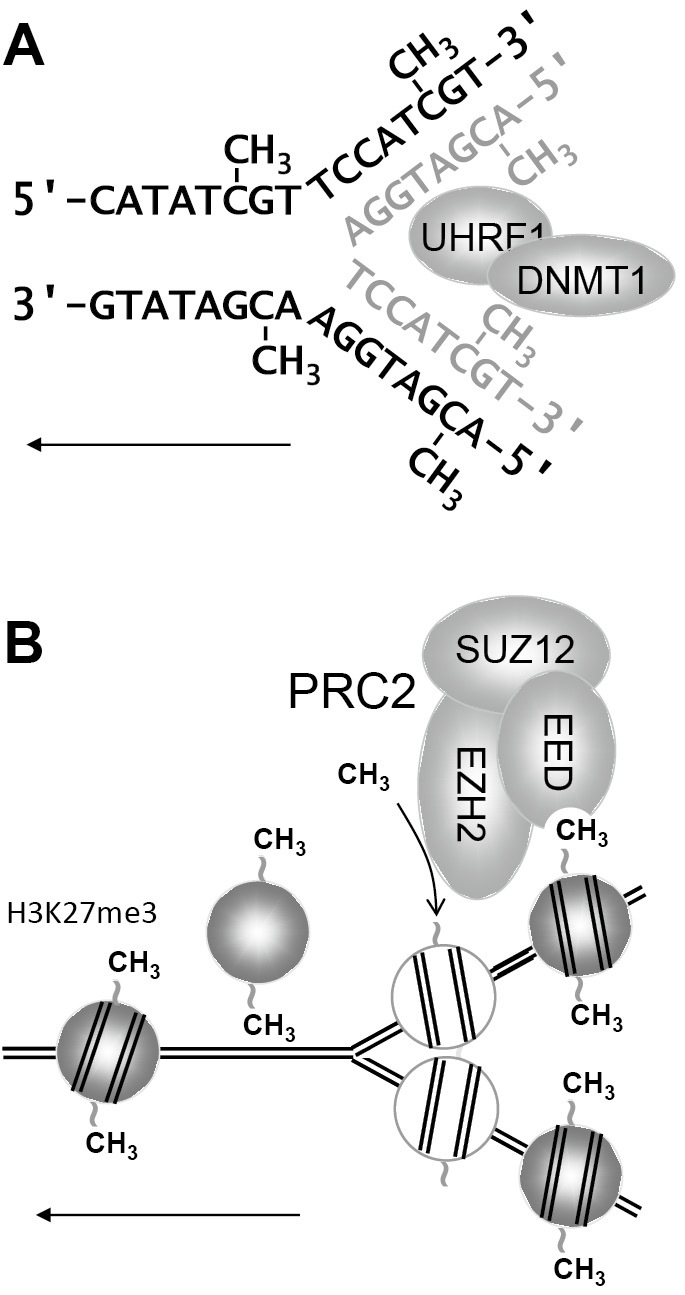
** Replication of the DNA methylation and H3K27me3**. A) UHRF1 (Ubiquitin Like With PHD And Ring Finger Domains 1) and DNMT1 (DNA methyltransferase 1) are recruited to the replication fork and recognize hemimethylated CpGs. DNMT1 methylates the CpG dinucleotides in the newly synthesized DNA. B) Nucleosomes are disassembled and reassembled on the two daughter strands, along with new histones. The Polycomb repressive complex (PRC2) copies H3K27me3 from old nuclesomes (grey balls) to new nucleosomes (white balls). PRC2 consists of EED (Embryonic ectoderm protein), which reads H3K27me3, EZH2 (Enhancer of zeste 2), which methylates H3K27, and SUZ12. H3K9me3 is read and copied by SUV39H1/2 (suppressor of variegation 3–9 homolog 1; not shown). Straight arrow, direction of movement of replication fork.

Among the many chromatin modifications known today, only the methylation of DNA, the methylation of histone H3 at lysine 9 (H3K9me3) and the methylation of histone H3 at lysine 27 (H3K27me3), all of which are hallmarks of repressive chromatin, can be replicated along with the DNA, because there are chromatin modifiers that can read and copy these modifications (Fig. 1). It is a matter of debate whether nucleosomes remember where they were before DNA replication [3]. Thus, strictly speaking, only DNA, H3K9 and H3K27 methylation (and possibly the position of nucleosomes) contribute to the cellular memory, although in common parlance all chromatin modifications are subsumed under the term “epigenetics”. Repressive chromatin does not only maintain the silent states of genes that have been switched off during development, but also serves to maintain the silencing of one allele in X inactivation and genomic imprinting.

## X inactivation and X-linked diseases

In female mammals, one X chromosome is inactivated to ensure that levels of X-linked genes are equal between XY males and XX females, although 15–30 % of X-linked genes appear to escape inactivation. In females with a normal karyotype, X inactivation is random with regard to the maternal and paternal X chromosome (the situation is different in the case of an X-autosome translocation). It is initiated at the X inactivation centre (Xic) [4], which harbours the *Xist* gene (*X-inactive specific transcript*). *Xist* RNA is transcribed from the X chromosome that has been selected to become inactivated and coats this chromosome. X inactivation involves an extensive change of chromatin modifications: active histone marks are removed and repressive histone marks are deposited. Later, DNA methylation plays an important role in maintaining the inactive state.

Since females are mosaics with cells in which the paternal X chromosome and cells in which the maternal X chromosome is inactivated, they are less susceptible to X-linked disorders. First, a pathogenic variant is not expressed in all cells. Second, cells expressing a pathogenic variant can receive the protein from cells that express the wildtype allele. Third, cell expressing a pathogenic variant may be outgrown by cells expressing the normal allele. Thus, the clinical manifestations of an X-linked pathogenic variant are milder or non-existent. However, there some X-linked diseases which are lethal in males and manifest in females.

## Genomic imprinting and imprinting disorders

The human genome harbors more than 100 genes that are expressed from either the paternal or the maternal allele only. These genes are subject to genomic imprinting, which is an epigenetic process by which the male and the female germ line confer a specific mark (mainly DNA methylation) onto these loci. Most of the imprinted genes occur in clusters, which are under the control of an imprinting control centre. In humans, clinically relevant imprinted gene clusters have been found on chromosomes 6, 7, 11, 14, 15, 16 and 20.

Loss or duplication of the one and only active allele by chromosomal deletions or duplications, uniparental disomy, genetic mutations and epimutations (see below) can lead to disease. At present, thirteen imprinting disorders have been recognized (abbreviation and chromosomal location in brackets): Transient Neonatal Diabetes Mellitus (TNDM, 6q24), Silver-Russell syndrome (SRS, 7 and 11p15), Birk-Barel syndrome (8q24.3), Beckwith-Wiedemann syndrome (BWS, 11p15), Kagami-Ogata syndrome (KOS14, 14q32), Temple syndrome (TS14, 14q32), Prader-Willi syndrome (PWS, 15q11q13), Angelman syndrome (AS, 15q11q13), Central precocious puberty (CPPB2, 15q11.2), Schaaf-Yang syndrome (SHFYNG, 15q11.2), Upd(16)mat syndrome (16), Pseudohypoparathyroidism (PHP1B,C,A; 20q13), Mulchandani-Bhoj-Conlin syndrome (MBCS, 20) [5]. Some patients with TNDM1 have abnormal DNA methylation at multiple loci (Multilocus imprinting disturbances; MLID).

## Epimutations

**Figure 2:**
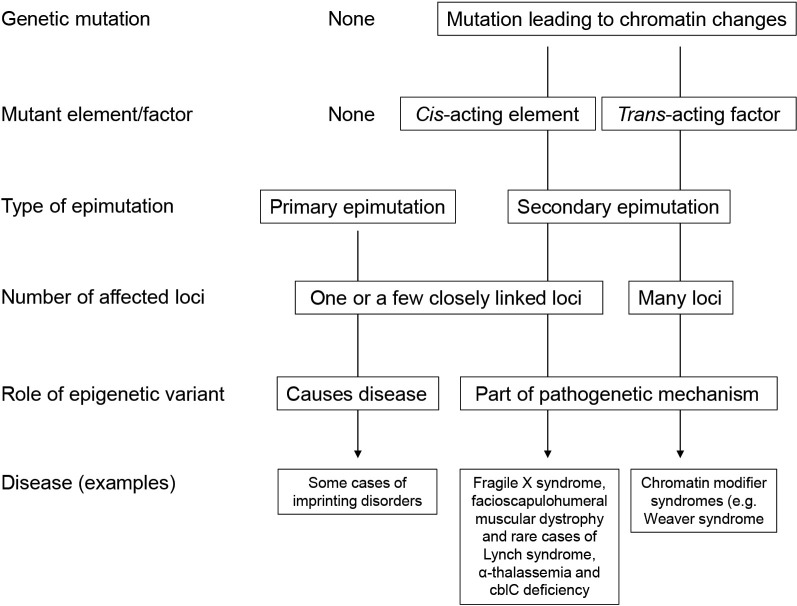
** Classification of epimutations**. Epimutations are primary or secondary epimutations. Primary epimutations are one of several causes of imprinting disorders. Other causes are a mutation in a *cis*-regulatory element or a *trans*-acting factor (see text). Fragile X syndrome and facioscapulohumeral muscular dystrophy are caused by the expansion or contraction of a tandem repeat sequence; above or below a certain number of repeats, the DNA is methylated or demethylated (→ secondary epimutation in *cis*). Weaver syndrome is an example of diseases involving secondary epimutations caused by a mutation in a *trans*-acting factor.

Aberrant chromatin states that are associated with a deleterious change in the kinetics of gene expression are called “epimutations”. An epimutation can be a primary epimutation that has occurred in the absence of any genetic change or a secondary epimutation that results from a mutation in a *cis*-acting regulatory element or *trans*-acting factor [6]. Epimutations may play a causative role in disease, for example in imprinting disorders, may be part of the pathogenetic mechanism as in the fragile X syndrome and in chromatin modifier syndromes or just reflect a disease state (Fig. 2).

## Epimutations in imprinting disorders

Epimutations in imprinting disorders result from an error in imprint erasure in a primordial germ cell, an error in imprint establishment in a maturing germ cell or an error in imprint maintenance in an early embryonic cell. Primary epimutations affecting an imprinted region are rare stochastic events and a direct cause of an imprinting disorder. Since they do not result from a genetic defect, they are not associated with an increased recurrence risk. Secondary epimutations result from imprinting center mutations affecting in *cis* the establishment or maintenance of an imprint. In familial cases, the recurrence risk is 50 %, but the penetrance depends on the sex of the mutation carrier. Mutations of the AS imprinting control center on chromosome 15, for example, which is necessary for the establishment of the maternal imprint in 15q11q13, is silently transmitted through the paternal germline, but causes AS when transmitted through the maternal germline [7].

Many cases of MLID are caused by a mutation in *ZFP57*, which encodes a transcriptional repressor. ZFP57 binds to the methylated hexanucleotide motif TGCmCGC in imprinting control regions to maintain DNA methylation after fertilization. MLID may also be caused by genetic variants of maternal effect genes such as *NLRP5*, which encode proteins supplied by the oocyte and required for early embryonic development, but this needs to be confirmed. Multi-locus imprinting errors are an example of secondary epimutations caused by a genetic defect in a *trans*-acting factor.

DNA methylation testing is a major component of the diagnostic work-up of patients suspected of having an imprinting disorder.

Apart from imprinting disorders, primary epimutations are very rare. Most of the epimutations in non-imprinting disorders are secondary epimutations caused by a genetic variant in *cis* or, more often, by a genetic variant in *trans*.

## Epimutations caused by a genetic variant in *cis*

Several genetic variants can cause an epimutation in *cis*. In this case, the epimutation is not the cause of the disease, but part of the pathogenetic mechanism. Here I discuss in more detail two diseases, in which an expansion and a contraction of a tandem repeat sequence affects the epigenetic state of a gene: the fragile X mental retardation syndrome 1 (FMR1) and the facioscapulohumeral muscular dystrophy (FSHD).

FMR1 is an X-linked disease caused by the expansion of an unstable trinucleotide repeat (CGG) within the promoter and exon 1 region of the *FMR1* gene [8]. The number of CGGs varies in the human population. Trinucleotide repeats with 55 and more copies are unstable and expand to several hundred copies during the proliferation of the diploid oogonia in the foetal ovary. Repeat expansions of up to 200 copies are called premutations. Premutations do not cause FMR1, but increase the risk for Fragile X Tremor Ataxia Syndrome (FXTAS) and Fragile X Premature Ovarian Insufficiency (FXPOI). After fertilization of an oocyte carrying more than 200 copies, the CGG repeat and the *FMR1* gene promoter are methylated. DNA methylation and the establishment of repressive chromatin in this region silence the FMR1 gene.

FSHD is an autosomal dominant disorder that has been linked to a 3.3 kb tandemly repeated sequence (D4Z4) in the subtelomeric region of the long arm of chromosome 4, each containing a copy of the double homeobox protein 4 (DUX4). In normal individuals the number of D4Z4 repeats varies between 11 and 150 units, and expression of *DUX4* is inhibited by DNA methylation. Patients with FSHD have fewer than 11 repeats and these are not methylated. Contraction of the repeats is accompanied by loss of DNA methylation and unsilencing of *DUX4* [9]. DUX4 is a pioneer transcription factor that acts on several target genes to cause FSHD. One form of FSHD (FSHD4) can also result from a mutation in *DNMT3B* (see Table 1).

There are also several examples in which a genetic mutation in one gene affects the epigenetic state of an adjacent gene. This has been observed in some patients with Lynch syndrome [10], α-thalassemia [11] and combined methylmalonic acidemia and homocystinuria, cblC type [12]. In these cases, transcriptional readthrough from a mutant neighboring gene has caused silencing and methylation of the disease gene.

## Epimutations caused by a genetic variant in *trans*

The epigenetic system comprises more than 100 proteins involved in making, reading and erasing chromatin modifications. Among them are four members of the DNA methyltransferase (DNMT) family, three Fe(II)/α-ketoglutarate-dependent dioxygenases (TETs), which erase DNA methylation, six methyl-CpG-binding proteins, >20 histone acetyltransferases (HATs) and other factors with HAT activity, 18 histone deacetylases (HDACs), 20 lysine methyltransferases (KMTs), eight lysine demethylases (KDMs), nine protein arginine methyltransferases (PRMTs), several other histone modifying enzymes (kinases, dephosphorylases, ubiquitinases, deubiquitinases etc.) and >20 nucleosome remodelling factors. A mutation affecting any of these factors leads to aberrant chromatin states and altered kinetics of gene expression at very many loci, although only some of these loci may be disease-relevant. As of 2023, more than 70 genetic syndromes caused by a mutation in a chromatin modifier have been recognized ([13] and Table 1). Depending on the gene and the function of the gene product, a mutation results in the loss or gain of function. The diseases follow autosomal dominant, autosomal recessive or X-linked inheritance, although most cases are sporadic.

Weaver syndrome is a good example of this class of diseases. It is caused by a mutation in the *EZH2* gene, which codes for a H3K27 histone methyltransferase, which is part of the Polycomb repressive complex 2 (PRC2; see Fig. 1 and Table 1). Mutations in the other two PRC2 components (EED and SUZ12) cause the “Weaver-like” Cohen-Gibson syndrome and Imagawa-Matsumoto syndrome, respectively (Table 1).

Chromatin modifiers cannot read DNA sequence context, but are recruited to specific genomic regions by transcription factors, which recognize and bind to their cognate sequence element. Pioneer factors can even bind to and open repressive chromatin. In fact, the genome-wide patterns of DNA methylation, histone modifications and nucleosome position are primarily shaped by transcription factors [14,15]. Mutations affecting transcription factors also affect the epigenome, because absent or dysfunctional transcription factors do not only fail to activate or repress their target genes, but also fail to recruit chromatin modifiers to their target loci. In these cases, aberrant chromatin states reflect rather than contribute to disease. Therefore, I do not cover transcription factor diseases in this review.

**Table 1:** ** Secondary epimutations caused by mutations affecting chromatin modifiers.** Note that many histone modifying enzymes also modify non-histone protein. Table reproduced and modified from [13] under the terms of the Creative Commons Attribution License from (© Fu, Merrill, Gibson, Turvey and Kobor). mC, 5-methylcytosine

Type of chromatin modification	Type of chromatin modifier	Gene	Target	Disease	OMIM
DNA methylation	DNA methyltransferases	*DNMT1*	Cytosine	Hereditary sensory neuropathy type IE (HSANIE)	# 614116
				Autosomal dominant cerebellar ataxia, deafness, and narcolepsy (ADCADN)	# 604121
		*DNMT3A*	Cytosine	Tatton-Brown-Rahman syndrome (TBRS)	# 615879
				Heyn-Sproul-Jackson syndrome (HESJAS)	# 618724
		*DNMT3B*	Cytosine	Immunodeficiency-centromeric instability-facial anomalies syndrome 1 (ICF1)	# 242860
				Facioscapulohumeral muscular dystrophy 4 (FSHD4)	# 619478
	Methyl-CpG-binding proteins	*MBD5*	mC	Intellectual developmental disorder, autosomal dominant 1	# 156200
		*MECP2*	mC	Rett syndrome (RTT)	# 312750
				Rett syndrome-associated severe neonatal encephalopathy	# 300673
				X-linked syndromic intellectual developmental disorder-13 (MRXS13)	# 300055
				X-linked Lubs-type syndromic intellectual developmental disorder (MRXSL)	# 300260
		*GATAD2B* (subunit of MECP1 complex)	mC	Gand syndrome	# 615074
		*ZFP57*	TGCmCGC	Transient neonatal diabetes mellitus 1 (TNDM1) and multilocus imprinting disturbance (MLID)	# 601410
	Methylcytosine dioxygenases	*TET2*	Modified cytosine	Immunodeficiency-75 (IMD75)	# 619126
		*TET3*	Modified cytosine	Beck-Fahrner syndrome (BEFAHRS)	# 618798
Histone modification	Lysine-specific methyltransferases (KMT)	*KMT2A/MLL1*	H3K4	Wiedemann-Steiner syndrome (WDSTS)	# 605130
		*KMT2B/MLL2*	H3K4	Dystonia 28, childhood-onset (DYT28)	# 617284
		*KMT2C/MLL3*	H3K4	Kleefstra syndrome 2	# 617768
		*KMT2D/MLL4*	H3K4	Kabuki syndrome 1	# 147920
		*KMT2E/MLL5*	H3K4	O’Donnell-Luria-Rodan syndrome (ODLURO)	# 618512
		*KMT2F/SET1A*	H3K4	Early-onset epilepsy with or without developmental delay (EPEDD)	# 618832
				Neurodevelopmental disorder with speech impairment and dysmorphic facies (NEDSID)	# 619056
		*KMT2G/SET1B*	H3K4	Intellectual developmental disorder with seizures and language delay (IDDSELD)	# 611055
		*KMT2H/ASH1L*	H3K36	Autosomal dominant intellectual developmental disorder-52 (MRD52)	# 617796
		*KMT1D/EHMT1/GLP*	H3K9	Kleefstra syndrome 1 (KLEFS1)	# 610253
		*EED*	H3K27	Cohen-Gibson syndrome (COGIS)	# 617561
		*EZH2*	H3K27	Weaver syndrome (WVS)	# 277590
		*SUZ12*	H3K27	Imagawa-Matsumoto syndrome (IMMAS) / SUZ12-related overgrowth	# 618786
		*KMT3A/SETD2*	H3K36	Luscan-Lumish syndrome (LLS)	# 616831
		*KMT3B/NSD1*	H3K36	Sotos syndrome (SOTOS)	# 117550
		*KMT3G/NSD2*	H3K36	Rauch-Steindl syndrome (RAUST)	# 619695
		*SETD5*	H3K36	Autosomal dominant intellectual developmental disorder-23 (MRD23)	# 615761
		*KMT5B/SUV420H1*	H4K20	Autosomal dominant intellectual developmental disorder-51 (MRD51)	# 617788
	Lysine-specific demethylases (KDM)	*KDM1A/LSD1*	H3K4me1/2, H3K9me1/2	Cleft palate, psychomotor retardation, and distinctive facial features (CPRF)	# 616728
		*KDM3B/JHDM2b*	H3K9me	Diets-Jongmans syndrome	# 618846
		*KDM4B/JMJD2B*	H3K9/ H3K36me2/3	Autosomal dominant intellectual developmental disorder-65 (MRD65)	# 619320
		*KDM5B/JARID1B*	H3K4me1/2/3	Autosomal recessive intellectual developmental disorder-65 (MRT65)	# 618109
		*KDM5C/JARID1C/SMCX*	H3K4me2/3	Claes-Jensen type of X-linked syndromic intellectual developmental disorder (MRXSCJ)	# 300534
		*KDM6A/UTX*	H3K27me2/3	Kabuki syndrome 2	# 300867
		*KDM6B/JMJD3*	H3K27me2/3	Neurodevelopmental disorder with coarse facies and mild distal skeletal abnormalities (NEDCFSA)	# 618505
		*KDM7B/PHF8*	H3K9	Siderius-type X-linked syndromic intellectual developmental disorder (MRXSSD)	# 300263
	Histone acetyltransferases (HAT)	*KAT3A/CREBBP*	H2A; H2B; H3	Rubinstein-Taybi syndrome (RSTS1)	# 180849
				Menke-Hennekam syndrome-1 (MKHK1)	# 618332
		*KAT3B/EP300*	H2A; H2B; H3	Rubinstein-Taybi syndrome 2 (RSTS2)	# 613684
				Menke-Hennekam syndrome-1 (MKHK2)	# 618333
		*KAT5/TIP60*	H4; H2A	Neurodevelopmental disorder with dysmorphic facies, sleep disturbance, and brain abnormalities (NEDFASB)	# 619103
		*KAT6A/MYST3/MOZ*	H3K9	Arboleda-Tham syndrome (ARTHS)	# 616268
		*KAT6B/MYST4/MORF*	H3K9	Genitopatellar syndrome (GTPTS)	# 606170
				SBBYS variant of Ohdo syndrome (SBBYSS)	# 603736
		*KANSL1*	H4K16	Koolen de Vries syndrome (KDVS)	# 610443
		*KAT8/MYST1/HMOF*	H4K16	Li-Ghorgani-Weisz-Hubshman syndrome (LIGOWS)	# 618974
		*BRPF1* (interacts with HATs)	H3K23	Intellectual developmental disorder with dysmorphic facies and ptosis	# 617333
		*TRRAP* (subunit of HAT complexes)		Deafness, autosomal dominant 75	# 618778
				Developmental delay with or without dysmorphic facies and autism	# 618454
	Histone deacetylases (HDAC)	*HDAC4*		neurodevelopmental disorder with central hypotonia and dysmorphic facies (NEDCHF)	# 619797
		*HDAC6*		X-linked dominant chondrodysplasia	# 300863
		*HDAC8*		Cornelia de Lange syndrome-5 (CDLS5)	# 300882
		*PHF21A* (subunit of BRAF HDAC complex)		Intellectual developmental disorder with behavioral abnormalities and craniofacial dysmorphism with or without seizures	# 618725
	Kinase	*RPS6KA3*	H3S10	Coffin-Lowry syndrome (CLS)	# 303600
				X-linked intellectual developmental disorder-19 (XLID19)	# 300844
	Ubiquitinase	*UBE2A*	H2B	Intellectual developmental disorder, X-linked syndromic, Nascimento type	# 300860
	Deubiquitinase	*BAP1*	H2AK119ub	Kury-Isidorsyndrome (KURIS)	# 619762
			H2AK119ub	Tumor predispositiom syndrome-1 (TPDS1)	# 614327
		*ASXL1*	H2AK119ub	Bohring-Opitz syndrome	# 605039
Histone type		*HIST1H1E*		Rahman syndrome	# 617537
Chromatin remodelling	SWI/SNF family – BAF complex	*ARID1B/BAF250B*		Coffin-Siris syndrome-1 (CSS1)	# 135900
		*ARID1A/BAF250A*		Coffin-Siris syndrome-2 (CSS2)	# 614607
		*SMARCB1/BAF47*		Coffin-Siris syndrome-3 (CSS3)	# 614608
		*SMARCA4/BRG1*		Coffin-Siris syndrome-4 (CSS4)	# 614609
		*SMARCE1/BAF57*		Coffin-Siris syndrome-5 (CSS5)	# 616938
		*ARID2/BAF200*		Coffin-Siris syndrome-6 (CSS6)	# 617808
		*DPF2/BAF45D*		Coffin-Siris syndrome-7 (CSS7)	# 618027
		*SMARCC2/BAF170*		Coffin-Siris syndrome-8 (CSS8)	# 618362
		*SMARCA2/BRM*		Nicolaides-Baraitser syndrome (NCBRS)	# 601358
				Blepharophimosis-impaired intellectual development syndrome (BIS)	# 619293
		*SMARCD1*		Coffin-Siris syndrome 11	# 618779
		*SMARCD2*		Specific granule deficiency	# 617475
	Other SWI/SWF family members	*HELLS/SMARCA6*		Immunodeficiency-centromeric instability-facial anomalies syndromes 4 (ICF4)	# 616911
		*ATRX*	H3.3	X-linked alpha-thalassemia/mental retardation syndrome (ATRX)	# 301040
				X-linked intellectual disability-hypotonic facies syndrome-1 (MRXFH1)	# 309580
	CHD family	*CHD1*		Pilarowski-Bjornsson syndrome (PILBOS)	# 617682
		*CHD2*		Developmental and epileptic encephalopathy-94 (DEE94)	# 615369
		*CHD5*		Parenti-Mignot neurodevelopmental syndrome (PMNDS)	# 619873
		*CHD7*		CHARGE syndrome	# 214800
		*CHD8*		Intellectual developmental disorder with autism and macrocephaly (IDDAM)	# 615032
	CHD family – NuRD/Mi-2 complex and associated proteins	*CHD3*		Snijders Blok-Campeau syndrome (SNIBCPS)	# 618205
		*CHD4*		Sifrim-Hitz-Weiss syndrome (SIHIWES)	# 617159
		*PHF6*		Börjeson-Forssman-Lehmann syndrome (BFLS)	# 301900
	ISWI family	*BPTF/NURF301*		Neurodevelopmental disorder with dysmorphic facies and distal limb anomalies (NEDDFL)	# 617755
	Human INO80 complex	*SRCAP*	H2A.Z	Floating Harbor syndrome (FLHS)	# 136140
				Developmental delay, hypotonia, musculoskeletal defects, and behavioral abnormalities (DEHMBA)	# 619595

## Episignatures

Microarray-based genome-wide DNA methylation scans of peripheral blood DNA have revealed unique DNA methylation patterns called “episignatures” in more than 60 genetic syndromes caused by mutations in genes encoding chromatin modifiers and transcription factors, although many of the affected proteins are not involved in methylating or demethylating DNA [16]. For an episignature it does not matter if the changes in DNA methylation cause disease, are part of the pathogenetic mechanism or just reflect the disease state. At individual CpG sites, the changes are small, which indicates that only a few cells are affected or that there is a change in the cell mixture distribution. Based on the methylation levels of ~150 CpGs that have been found to be the most representative CpGs for a given disease, disease-specific diagnostic classifiers have been developed, which can aid the diagnosis of a rare disease and the classification of variants of unknown significance (VUS).

## Conclusions and Outlook

Aberrant chromatin states (epimutations) play an important role in a number of rare diseases. For several diseases, DNA methylation analysis can help to confirm or exclude a clinical diagnosis. In diseases caused by a mutation in a chromatin modifier it remains to be elucidated which are the disease-relevant loci that are affected by a secondary epimutation and how the dysregulation of these loci leads to disease. It also remains to be shown whether newly developed tools such as epigenome editing with modified CRISPR-Cas enzymes, which, for example, allow to add or remove methyl groups to and from CpG dinucleotides in specific gene regulatory regions, can be developed into therapeutic approaches [17]. In view of the fact that only a few chromatin modifications are mitotically stable (see Introduction) and that cell fate decisions made during early development cannot be undone in childhood or adulthood, I am rather skeptical about the clinical application of this approach in rare diseases. Probably, other avenues have to be explored.
